# Bread Enrichment with Oilseeds. A Review

**DOI:** 10.3390/foods7110191

**Published:** 2018-11-20

**Authors:** Beatriz de Lamo, Manuel Gómez

**Affiliations:** Food Technology Area, College of Agricultural Engineering, University of Valladolid, 34004 Palencia, Spain; beatriz.de.lamo.santamaria@gmail.com

**Keywords:** bread, fortification, sunflower, flaxseed, chia, sesame, mucilage

## Abstract

The use of oilseeds in bakery products has gained popularity in recent years, both for their organoleptic and nutritional characteristics. The aim of this work is to provide an overview of the studies centered on the use of oilseeds (flaxseed, chia, sunflower, pumpkin, sesame and poppyseed) in breads and other bakery products. This review highlights the effect of oilseeds on the mechanical and physical properties of bread according to the enrichment level, origin and way of addition (whole, crushed, oil or mucilage). In general, the incorporation of oilseeds improves the nutritional profile of bakery products with and without gluten, and provides several health benefits. Mucilages of oilseeds can also act as a fat replacer thanks to their properties. The incorporation of oilseeds modifies the rheology of the doughs, the volume of the products and their texture, affecting their organoleptic characteristics and their acceptability. Nevertheless, these changes will depend on the type of seed used, as well as on the method of addition.

## 1. Introduction

Oilseeds are grains with a high amount of oil. They have been part of the human diet for a long time, but it has only been in the last few decades that their use and production have trended upward. The use of oilseeds in the modern market has increased due to the growing number of people concerned about a healthier lifestyle, as well as better knowledge of their attractive composition [1]. The chemical composition of oilseeds depends on their growing environment, genetics and processing conditions [2]. Generally, these seeds have lower carbohydrate content and higher protein content than cereals, high levels of fiber, and omega-6 and omega-3 essential fatty acids [3]. These seeds also enclose a high proportion of natural antioxidant compounds (tocopherol, beta-carotene chlorogenic acid, caffeic acid and flavonoids), vitamins and minerals [4,5,6]. Moreover, the absence of gluten in these seeds allows their ingestion by celiac disease sufferers [1,6]. Thanks to this composition, some of these seeds have proven effective in controlling and preventing metabolic diseases (hypertension, hypercholesterolemia, diabetes, coronary heart disease and several types of cancer) as well as providing interesting properties to foods (body, texture and taste improvement) [5,6,7,8]. 

Since bread is considered a staple food worldwide because of its nutritive value, low price and its simplicity of use, it is ideally suited for fortification with oilseeds. The impact of oilseeds on bread baking has been studied in many investigations. Oilseeds can be incorporated whole, crushed and as pressed oil, and their beneficial effect on health seems to depend on the method of addition [9]. Further, changes in dough rheology and the quality of final products, including their sensory acceptability, depends on whether they are incorporated whole or crushed. Other aspects, such as the oxidative stability of the enriched products, must be also considered [10]. In the case of chia and flax, the mucilaginous polysaccharide that these seeds exude when they are placed in aqueous medium can also be added to the formulation to provide advantageous technological properties in terms of food development. The hydration of grains or flours before their incorporation into the bread doughs, in order to extract these polysaccharides, can modify the rheology of the doughs and the quality of the breads [11]. These grains can also be added only on the surface of breads or other products, in which case the influence on the final product will be less. Differences in the development of breads have been reported based on the method of incorporation and the pretreatment of the oilseeds, among other things, and therefore there are still numerous challenges to solve in their use (oxidation of the lipids, the impact of the gluten network to the structure, etc.). 

Despite the wide use of oilseeds in bakery products, scientific studies on them are scarce. The purpose of this review is to deepen the knowledge of the use of different oilseeds for the development of enriched breads, both from a nutritional and organoleptic point of view.

## 2. Nutritional Profile

Oilseeds, as their name suggests, have a high fat content that usually exceeds 40%, except for chia and pumpkin seeds, which have a lower percentage (Table 1). The low values of oil content in pumpkin seeds reported by the United States Department of Agriculture (USDA) may conflict with some varieties analysed by Stevenson et al. [8]. In their research, some varieties of pumpkin seeds were 30% lipids, while in the study by Seymen et al. [7] the oil contents of pumpkin seeds were between 33% and 47% depending on the variety. Furthermore, these fats stand out for their low level of saturated fatty acids and for their high content of oleic acid in sesame seeds; linoleic acid (ω6) in sunflower, sesame and poppy seeds; and linolenic acid (ω3) in flax and chia seeds. In the case of pumpkin seeds, apart from their high linoleic acid level, their oleic acid content is also highlighted [7,8,12]. These seeds also have significant protein content (between 15 and 20%) and a fiber percentage higher than that of cereal grains, with fiber content of flax and chia seeds over 25%. In the case of chia, different studies have reported higher values of fiber (between 35–40%) depending on the variety, and of which more than 85% is insoluble fiber [13,14,15].

Mineral content is noteworthy. The high calcium content of sesame, chia and poppyseed; the iron content of sesame and poppyseed; the zinc content of pumpkin; and the magnesium, phosphorus and potassium contents of most of these seeds stand out, and are higher than in wheat. As a negative aspect, sunflower seeds have high sodium content. They also stand out for their high level of vitamins B and E. In particular, sesame is notable for its high levels of vitamin B6 and folates; flaxseed for its high level of thiamine and folates; chia for its high level of niacin; and sunflower for its high content of niacin, vitamin B6, folates and vitamin E. Some of these seeds also show a high content of antioxidants. Thus, chia has a high concentration of polyphenols derived from caffeic acid and other antioxidant substances [13,14,16,17]. The antioxidant potential of flax [18], sesame [19,20], pumpkin [7] and sunflower seeds [21] has also been highlighted. In the case of chia and flax seeds, it has been demonstrated that the partial germination of the grains can increase both their antioxidant capacity and the content of phenolic compounds [22].

The interesting composition of these grains has attracted interest for their possible health benefits. Fiber consumption has been shown to be effective against CVD (Cardiovascular diseases), CVD mortality, coronary artery disease and several kinds of cancer [23], as well as against various gastrointestinal disorders, including: gastroesophageal reflux disease, duodenal ulcer, diverticulitis, constipation, and haemorrhoids [24]. The consumption of omega-3 fatty acids has also been associated with a reduction of cardiovascular morbidity and mortality, and dietary supplementation may also benefit patients with dyslipidaemia, atherosclerosis, hypertension, diabetes mellitus, metabolic syndrome, obesity, inflammatory diseases, neurological/neuropsychiatric disorders and eye diseases [25]. Lignans, a phytoestrogen that stands out among antioxidant substances, are found in important quantities in flax, sesame and pumpkin seeds and have shown promise in reducing growth of cancerous tumours, especially hormone-sensitive ones such as those of the breast, endometrium, and prostate [26]. Moreover, a synergistic effect between sesame lignans and the vitamin E activity of tocopherols has been proved. Vitamin E activity increases in the presence of these lignans, while the cholesterol-lowering effect of lignans is enhanced [27]. The stability of flax lignans to the conditions of baking processes and storage has also been demonstrated and thus no loss of functionality when incorporated into bakery products has been observed [28]. Likewise, it is known that chia helps to reduce postprandial blood glucose, the level of high-density lipoprotein in serum and diastolic blood pressure [29]. Moreover, different reviews suggest that the consumption of chia could help to increase the satiety index, prevent cardiovascular diseases, inflammatory and nervous system disorders, and diabetes, among others [6,30]. Flax seeds have shown a positive effect on cardiovascular diseases and hypercholesterolemic atherosclerosis [31] and the sesame consumption has been linked to positive effects against some illnesses such as cancer, oxidative stress, cardiovascular disease, osteoporosis and other degenerative diseases [32,33]. In the case of flaxseed, it has been demonstrated that the incorporation of the flour of this seed in a wide number of products does not affect the consumption preferences of the same by the population, so its use can be a good strategy to provide significant health-related benefits to patients with cardiovascular disease [34].

Many studies have focused on the nutritional enrichment of bakery products through the incorporation of this type of seed or by-products, such as oils, proteins or other derivatives. In general, an increase in the content of nutrients present in the seeds, or their derivatives, is confirmed, since there are no losses or significant changes in them during the baking process. As for the incorporation of proteins extracted from these seeds, Mansour et al. [35] observed that up to 22% replacement of wheat by pumpkin protein isolate is possible without adverse effects on bread quality. They also reported that the obtained breads have a higher level of protein, lysine and minerals, improving also the essential amino acid index. However, mineral enrichment was greater when the seed flour was incorporated. The study of El-Soukkary [36] confirmed these observations with isolated pumpkin seed protein and observed that the in vitro protein digestibility also improved. These effects are repeated with the incorporation of sesame proteins, but in this case, the maximum level of substitution was 18% [37]. The incorporation of sunflower seeds at levels of up to 16% allowed an increasing of the levels of tocopherol and certain minerals, such as Ca, Mg and Zn in normal and whole grain breads, as well as enriching them in fats with a high linoleic content [38]. The incorporation of chia, as seed or flour, increased the fiber content and omega-3 acids both in wheat breads and in gluten-free breads [39,40]. Some researchers have also studied the inclusion of chia flour up to 14% and have observed an increase in the levels of ash, protein, fiber and lipids [41]. Regarding the incorporation of flax, as seed or flour, the incorporation of milled brown flaxseed in breads at levels of up to 13% resulted in an increase in the content of fiber, P, K, Mg, Zn and linolenic (omega-3) [42]. These same authors observed a decrease in the total cholesterol, low-density lipoproteins (LDL) and the level of triglycerides in rats when they were fed with these breads compared to those fed with breads without addition of flaxseeds. On the other hand, the incorporation of ground flaxseed hulls at levels lower than 5% was sufficient to increase significantly the antioxidant activity in breads [43].

In general, the incorporation of these seeds, whole or ground, increases the content of fiber, proteins, minerals, omega-3, omega-6 and minerals. For this reason, the inclusion of oilseeds in gluten-free breads is of special interest, since it is known that the gluten-free diet can be low in fiber and minerals (iron, zinc, magnesium and calcium), and may contain excess saturated fats [44,45,46,47]. In fact, supplements of these micronutrients have been proposed to improve the quality of the gluten-free diet [48]. In general, gluten-free breads also have lower protein content and lower levels of certain minerals than wheat varieties [49,50,51,52]. Additionally, it must be considered that the incorporation of these seeds, together with ingredients such as milk powder in gluten-free breads, not only increases the mineral content of gluten-free breads, but can also increase their bioavailability, depending on the mineral and the starting flour [53]. In addition, several studies with rats have demonstrated the potential of gluten-free breads enriched with this type of seed to reduce the levels of triglyceride and improve the antiradical properties of serum [54].

Despite the clear evidence of the nutritional advantages of incorporating this type of seed in breads, the health benefits can sometimes depend on the method in which they are incorporated. Kuijsten et al. [9] showed that milling the flaxseeds improved the bioavailability of the enterolignans, and therefore their nutritional advantages increased. Similarly, the reduction of the particle size by wet micronisation increased the oral bioavailability of lignan glycosides from sesame meal [55]. However, when flaxseed products are incorporated into muffins or breads, the mammalian lignan production of flaxseed precursors depends on the time and dose, but not on the processing [56]. Although more research is needed in this regard, we must also consider the effects of this processing on the rheology of the doughs and the organoleptic quality of the breads. The stability of these products must also be considered during storage, something of great importance for industrial use. Although Malcolmson et al. [57] observed good stability of the milled flaxseed stored at room temperature in paper bags with plastic liners during a four-month period, other studies have shown that when the particle size decreases, the oxidative stability of these products decreases too [58]. Therefore, when milled oil seeds are used, aspects such as particle size and packaging conditions must be considered in order to maximise the stability of these products.

## 3. Flaxseed and Chia Seed Mucilages 

Chia seeds and flaxseeds are characterised by being very rich in mucilage. The mucilage of flaxseeds, consisting mainly of water-soluble polysaccharides, has a great water absorption capacity and rheological properties similar to those of guar gum [59]. In fact, flaxseed mucilages have been proposed as a substitute for gluten in the preparation of gluten-free breads in order to improve their acceptability against a mixture of pectin and guar gum [60]. Its composition includes a neutral fraction, composed of arabinoxylans, and an acid fraction composed of polysaccharides similar to pectin [61]. As with flax mucilage, chia mucilages are extracted in water, more easily if the temperature is high; when the proportion of water-seed increases, the saline content of the water decreases and the pH increases [1], so the conditions of the mixture can affect the interactions with this component. In this research, the hydration manages to extract mucilage with yields of 7%, somewhat lower than the maximum achieved with flax, which was slightly higher than 9% [59]. The chia mucilage has also been compared with guar gum and locust bean gum because of its high hydration capacity [62] and great thickening power [63]. Its structure corresponds to that of arabinoxylans, but with greater amounts of uronic acids than those of flax, imparting anionic characteristics to the macromolecule [64]. On the other hand, its ability to increase the stability of oil-in-water (O/W) emulsions has been demonstrated [65]. In the case of flax mucilage, its good foam stability properties in aqueous solutions have also been demonstrated [66].

Just like other hydrocolloids, the use of chia mucilage as a stabiliser in ice cream [67] and as a substitute for fats in breads, cakes [68,69,70] or mayonnaise [71] has been proposed. The use of hydrocolloids with high water absorption capacity and thickening power, such as xanthan gum, in wheat bread increases the absorption of the doughs, the development time and stability in the kneading, generates more tenacious and less extensible doughs, and can increase the specific volume and the firmness of the breads [72]. Similarly, guar gum has proved efficient in improving the volume of breads made with non-frozen and frozen dough [73]. This type of hydrocolloid has also been proposed to reduce water losses in storage, both of finished breads [74] and doughs and breads under refrigeration [75]. Usually, gluten-free formulations have applied hydrocolloids to mimic the viscoelastic properties of gluten [76]. Among these hydrocolloids, those with high gas-holding capacity and high thickening power, such as guar gum or xanthan gum, have been suggested [77], and are some of the most widely used industrially. At a nutritional level, these gums are also interesting. Guar gum is capable of significantly decreasing the in vitro hydrolysis of starch, an effect that has been attributed both to guar galactomannan, which acts as a physical ‘barrier’ to alpha-amylase-starch interactions, and to the effect of guar gum on digesta viscosity [78]. Likewise, breads enriched with arabinoxylans can help control the glycaemic level in diabetics [79]. However, the incorporation of chia mucilage has only been able to reduce the glycaemic index estimated in the crust of pita breads, but to increase it in the crumb [80]. More research would be necessary but, in general, the use of chia and flax mucilage can be very interesting in baking, isolated or together with the flour or seeds, in which case its functionality will depend on hydration and its release from the original matrix to interact with the components of the dough.

## 4. Dough Rheology

The rheology of bread doughs gives us an idea of how they behave in different processes, and can also help us to predict some final characteristics of the breads. Current studies on the influence of oleaginous seeds and their flours on the rheology of bread doughs are limited, but we expect that there is an influence depending on their composition, since they are rich in oil, vegetable proteins and fiber. Thus, it is known that the addition of small amounts of oil or fat leads to greater flexibility and workability of the doughs, greater final volume, a finer alveolar structure, and a softer final texture [81]. The addition of fats and oils also delays starch retrogradation, prolonging the shelf-life of the breads. On the other hand, the vegetal proteins modify the farinographic properties in a different way according to their type [82,83], but in general they reduce the strength of the doughs and their extensibility. The incorporation of fiber usually increases water absorption, the mixing tolerance and the tenacity of the doughs, but reduces extensibility [84]. The type of incorporation must be also considered since, if oilseeds are incorporated as flour, their different components will interact with the components of the dough, will compete with them, or will establish synergies. However, if they are incorporated as seeds, although these seeds can also have an effect on the properties, their components will not interact since they will remain inside the seed coat.

In the farinographic analysis of doughs with chia at 5%, the incorporation of seeds does not modify the behaviour during the kneading but decreases absorption slightly [85]. However, in this same research the addition of whole or low-fat chia flour increased the stability of the doughs, and when defatted chia flour was added the absorption also increased. Contrary to what was observed in this study, Steffolani et al. [11] reported that a 5% addition of chia flour does not affect absorption, but it increases with a 15% addition. The incorporation of chia also reduced the stability of the doughs and increased the development time. The decrease in the stability of the doughs matches the observations of Koka and Anil [86] with the incorporation of flaxseed, Moreira et al. [87] with chia flour, and Matthews et al. [88] with the addition of different oilseed flours. A greater absorption of water was also observed with the incorporation of these flours in all these studies. In general, there seems to be agreement that the use of these types of flours does not modify the absorption or increase it, while the effect on stability is contradictory. The differences may be due to the different starting flours, the percentage and the type of oilseed flour used. In fact, the wheat flour used by Iglesias, Puig and Haros [85] presented lower stability values than those used by Steffolani et al. [11] or Koka and Anil [86], making it easier to increase this stability. Regarding the variety of seed used, Svec et al. [89] even observed that using different types of chia (white or black) modified the effect of these flours on the amylographic analysis, although, in both, the peak viscosity increased, the viscosity rise was faster and the pasting time decreased. These results coincide with what Verdu et al. [90] observed by Rapid Visco Analyser (RVA), which has been attributed to the presence of mucilage. The incorporation of flax and chia flours also tends to reduce the extensibility of the doughs and the energy of deformation, measured with the extensograph or with the alveograph [11,86], which can cause problems in the use of the doughs or in the retention of gas during fermentation. In fact, Steffolani et al. [11] observed that the gluten network of the doughs with 10% of seeds or chia flour was broken before the control dough, with gas escaping to the outside. This effect could be expected due to the dilution of the gluten located in the mixtures and responsible for these properties. Similar results have been observed due to the incorporation of lipids [91] and products with a high content of non-gluten-forming proteins [92], which can inhibit the formation of the gluten network.

In the case of gluten-free doughs, the use of equipment such as the alveograph or the extensograph is not possible because they are based on the measurement of the characteristics that the gluten confers on the doughs, such as extensibility or tenacity. It is usual to analyse the gluten-free doughs by conventional rheology, such as in oscillatory tests. There is limited research related to this, but Moreira et al. [87] observed that the incorporation of chia flour into doughs of chestnut flour reduced both the elastic and the viscous component. These results conflict with the observations of Zettel and Hitzmann [93] in doughs of sweet bread, but in this study the chia flour was used to replace fats together with a greater incorporation of water, so the changes in the rheology cannot be attributed exclusively to the incorporation of chia flour.

The previous hydration of chia flours or seeds can also have a great impact on the rheological properties of the doughs, since it allows the mucilage of this seed to flow into the water and interact better with the rest of the ingredients of the dough. Thus, the prehydration of the chia flour allows an increase of the absorption of doughs and reduces development times, although it does not modify either the alveographic properties [11] or the pasting properties of gluten-free flours [94].

In general, the modification of the rheology of the doughs with the incorporation of oilseeds or their flours can be confirmed. This modification seems to be more pronounced in the case of flours than of seeds although these changes are not very considerable if the amount of flour does not exceed 5%. The modification of the rheology of the doughs really depends on many factors, so one cannot draw general conclusions and each case must be studied.

## 5. Bread Quality

The effect of oilseed flours on breads is determined by their components. It is known that the incorporation of fibers [84], such as celluloses, or vegetable proteins [95] usually reduces the specific volume of the bread’s original firmer textures. The effect of the incorporation of oils on breads depends on their added quantity. In small amounts and depending on the type of oil added the incorporation of oils can be beneficial, while in larger quantities they weaken the dough and have negative effects on the volume of the breads [96]. Therefore, depending on the added product (wholemeal, protein concentrate, defatted flour), its composition and the amount added, the effects on the quality of bread may vary. Generally, a negative effect on the volume of the breads is expected after incorporation. When non-ground whole seeds are used, a minor effect is expected since not all of their components interact with the wheat flour.

Chia flour is one of the most studied for baking processes. In general, wheat breads reduce their volume, increase hardness and present darker crumbs as the level of chia flour increases, as some research on bread fortification [11,97] or the substitution of saturated fats for chia flour [39] has shown. However, some studies have reported an increase in the volume and a reduction of the hardness of breads with less than 10% chia flour [85,90]. These differences may be due to the wheat flours used, the formulations used or the time of fermentation. In fact, the fermentation time was optimised in these two studies compared to the previous ones, which used a fixed fermentation time. This would indicate that the doughs enriched with chia flour would not support an excess of fermentation. Thus, the drop in volume and the consequent increase in the hardness of the breads may be due to the loss of strength of the dough and the breakage of the gluten network during fermentation, as shown by Steffolani et al. [11]. Other research, focused on other oilseeds such as sesame [37] or pumpkin [35] at levels above 14%, showed a decrease in the volume of the breads when they were enriched with flours of these seeds or protein concentrates made from them. However, Koca and Anil [86] did not observe differences in the volume of breads enriched with flaxseed flour at levels between 5% and 20%, which the authors attributed to the functionality of flaxseed gum. Likewise, Skrbic and Filipcev [38] did not observe differences either between the specific volumes or between the hardness of the breads, both white and whole, made with sunflower seeds. The differences with other investigations may be due to the incorporation of seeds and not flours, so the interaction of the components of the seeds with the components and the gluten is lower. However, a certain relaxation of the doughs was observed in this study since a decrease in the height/width bread ratio of the breads with 12% addition was observed. In general, the addition of oilseeds seems to have a smaller effect on the volume of the breads than the addition of oilseed flours. The incorporation of oilseeds as flour can have a positive effect or not modify the volume of the breads if they are incorporated in small proportions, while it shows a negative influence on the volume when the quantities exceed a certain level. This was exactly what Mentes et al. [98] observed in a study on the enrichment of wheat breads with ground flaxseed, where the negative effects began to be noticed after adding 20% flaxseed, and an increase of the volume with levels of 10% flaxseed. Similarly, Sayed-Ahmad et al. [99] observed that the incorporation of chia seed powder (full fat) at levels between 4–6% did not modify the hardness of the whole breads, while its inclusion in cakes (defatted venues) even reduced it.

Volume is one of the most reliable qualities of bread. Bread loaves with high volume often attract consumers. To improve the volume of oilseed-enriched breads, the addition of vital gluten [97] or the prehydration of the oilseeds has been proposed. When oilseeds are prehydrated, their mucilage interacts with the rest of the ingredients of the dough as a hydrocolloid would to reinforce the dough [11]. For the same reason, the use of additives (emulsifiers such as diacetyl tartaric acid ester of mono- and diglycerides (DATEM), or oxidants such as ascorbic acid) to strengthen the doughs could be tested. Zettel and Hitzmann [93] managed not to modify the volume of the breads and reduce their hardness when they used chia flour to substitute fats by increasing the moisture content of the formulation when the flour was incorporated.

The results also seem contradictory for gluten-free breads, something usual in this type of research since they depend on the flour/starch used as a base, the substitute for gluten and the hydration. It is known that the incorporation of insoluble fibers [100] and proteins [101] usually reduces bread volumes. In the case of oil, it can have a beneficial effect by reducing the consistency of the batters, which usually increases the volume of the bread to a certain limit [102]. All these investigations have been carried out on gluten-free breads with hydroxypropyl methylcellulose (HPMC), which can reach volumes similar to wheat breads and are very sensitive to changes in the formulation. In general, research shows a decrease in the volume of the breads and an increase in the hardness when chia flour was incorporated [41,94], although Constantini et al. [40] did not observe significant differences when they incorporated this flour to 10% in bread with buckwheat flour. However, in this study, no gluten substitute was used and the specific volume obtained was less than 1.5 mL/g, so that the doughs barely increased during the fermentation and baking processes. In contrast, the investigation of Sandri et al. [41] showed specific volumes of 1.7 mL/g with xanthan gum and carboxymethylcellulose, and specific volumes of 6 mL/g using HPMC were reached in the research of Steffolani et al. [94], which showed a greater reduction in volume with the incorporation of chia flour. Thus, changes are more appreciated when the specific volume of the control bread is greater. 

The shelf life of enriched breads is another important aspect to consider. Although it has barely been studied in the research carried out until now, it is known that loaves with lower volumes and higher hardness generally harden more quickly [103]. Thus, the reduction of the volume of the loaves due to the incorporation of these seeds can accelerate the staling of the bread. Nevertheless, it is also known that lipids reduce the retrogradation phenomena of amylopectin [104] and, therefore, the rate of hardening of the loaves [105], so when oilseed flours are incorporated and the specific volume of the loaves is maintained, it is possible that the hardening of the loaves is delayed. It has been shown that the incorporation of chia flour can delay the retrogradation of amylopectin, responsible for the staling of the loaves [85]. The incorporation of ground flaxseed also retarded bread staling [98]. However, in long-life breads the oxidation of these lipids and their effect on the oxidation of other lipids present in the product must be considered, especially when they are incorporated as flour. Thus, breads enriched with flaxseed meal presented a higher level of peroxides after 8 weeks of storage [106]. An accelerated lipid oxidation, particularly polymerization compounds, was shown in chia-enriched biscuits [107]. These problems can be partially solved with the incorporation of antioxidant substances [108]. However, the possible combinations should also be considered since sesame seed extract has been shown to be effective in protecting oils from oxidation [109], and sesame oil has also been shown to be effective in delaying rancidity in oil of sunflower [110].

## 6. Sensory Properties

Although it has been proved that oilseeds can provide important health benefits, breads enriched with these seeds can only become commercially viable if consumers value their organoleptic properties. On this matter, multiple studies have evaluated the consumer acceptance of oilseed-enriched breads. In general, the oilseed enrichment of breads with and without gluten does not reduce the quality and acceptability to customers. Despite the addition of some seeds resulting in changes in the texture, colour and odour of the bread, there were no significant differences for crumb colour, crumb texture, quality and overall acceptability between the control and breads prepared with chia [11], sesame [37], pumpkin [36] and flax seeds [86]. 

As for breads enriched with oilseed flours, Verdú et al. [111] reported that bread enriched with 5% chia flour did not show significant sensory differences in respect to the control bread. This may be due to the low percentage of chia flour used. Coelho and Salas-Mellado [39] observed that the acceptability of enriched bread with oilseed flour is better than that of bread enriched with seeds, which they attributed to the higher specific volume of the bread with oilseed flour. However, in this research, they compared breads with 7.8 g/100 g of chia flour versus breads with 11 g/100 g of chia seeds, so the amount of chia could influence the evaluation of the consumers. Regardless, the use of prehydrated oilseed flours could be an option to improve the sensory properties of breads [11], probably due to the higher moisture level and less sensation of dryness, by releasing the mucilage prior to baking and increasing water retention during baking. In this respect, more research would be necessary in order to know the level of enrichment at which changes in the sensory properties become significant, since no agreement has been reached. The influence of enriched gluten-free breads with chia has been studied by authors such as Steffolani et al. [94] and Sandri et al. [41]. In both studies, no significant differences in terms of overall acceptability were reported in formulations with up to 15 g/100 g of chia flour or seeds.

In general, it can be affirmed that although the organoleptic characteristics of the breads change with the inclusion of oilseed seeds or their flours, there is no difference in the acceptability to consumers of these breads up to levels of 10–15%.

## 7. Conclusions

The incorporation of oilseeds improves the nutritional profile of bread, increasing its protein, fiber, vitamins, minerals, essential fatty acids and bioactive compounds. The use of oilseed mucilages has also been used successfully to replace the fat to produce healthier good quality breads. Therefore, the inclusion of these compounds in bakery products is of great interest, both in wheat products and in gluten-free products. However, it must be borne in mind that this inclusion modifies the rheology of the dough, depending on the way in which it is made (flour or seeds, prehydration or not) and the percentage used. Based on this, problems can result in changes to the final characteristics of the bread. To ensure the commercial success of these inclusions, it is necessary to consider the acceptability to consumers, which may vary depending on the type of inclusion and its percentage. In general, however, it seems that seed levels up to 15% can be obtained with good acceptability.

## Figures and Tables

**Table 1:** Nutrient content of some whole oilseeds (per 100g).

		Wheat	Sunflower	Flaxseed	Sesame	Chia	Pumpkin	Poppy
Nutrients	Water (g)	12.42	1.2	6.96	4.69	5.8	4.50	5.95
	Energy value (kcal)	332	582	534	573	486	446	525
	Protein (g)	9.61	19.33	18.29	17.73	16.54	18.55	17.99
	Total lipid (fat) (g)	1.95	49.8	42.16	49.67	30.74	19.40	41.56
	Carbohydrate (g)	74.48	24.07	28.88	23.45	42.12	53.75	28.13
	Fiber (g)	13.1	9	27.3	11.8	34.4	18.4	19.5
	Sugar (g)	1.02	2,73	1.55	0,30	N	N	2.99
Minerals	Calcium (mg)	33	70	255	975	631	55	1438
	Iron (mg)	3.71	3.8	5.73	14.55	7.72	3.31	9.76
	Magnesium (mg)	117	129	392	351	335	262	347
	Phosphorous (mg)	323	1115	642	629	860	92	870
	Potassium (mg)	394	850	813	468	407	919	719
	Sodium (mg)	3	655	30	11	16	18	26
	Zinc (mg)	2.96	5.29	4.34	7.75	4.58	10.30	7.9
Vitamins	Vitamin C (mg)	0	1.40	0.6	0	1.6	0.3	1
	Thiamin (mg)	0.297	0.106	1.644	0.791	0.62	0.034	0.854
	Riboflavin (mg)	0.188	0,246	0.161	0.247	0.17	0.052	0.1
	Niacin (mg)	5.347	7.04	3.08	4.515	8.83	0.286	0.896
	Vitamin B6 (mg)	0.191	0.804	0.473	0.79	N	0.037	0.247
	Folate (µg)	28	237	87	97	49	9	82
	Vitamin E (mg)	0.53	26.1	0,31	0,25	0,50	N	1.77
Lipids	Saturated (g)		5.219	3.663	6.957	3.33	3.670	4.517
	Monounsaturated (g)		9.505	7.527	18.759	2.309	6.032	5.982
	18:1 (g)		9.399	7.359	18.521	2.203	5.985	5.864
	Polyunsaturated (g)		32.884	28.73	21.773	23.665	8.844	28.569
	18:2 (g)		32.782	5.903	21.375	5.835	8.759	28.295
	18:3 (g)		0.069	22.813	0.376	17.830	0.077	0.0273

Source: United States Department of Agriculture (USDA) National Nutrient Database for Standard Reference (2018). N, there is no reliable information about the quantity of the nutrient.
